# Different Complement Activation Pathways Underly Cognitive Impairment and Type 2 Diabetes Mellitus Combined With Cognitive Impairment

**DOI:** 10.3389/fnagi.2022.810335

**Published:** 2022-03-08

**Authors:** Zhenxing Li, Weiwei Zhang, Feng Gao, Qiqiang Tang, Dongmei Kang, Yong Shen

**Affiliations:** ^1^Department of Neurology and Institute on Aging and Brain Disorders, The First Affiliated Hospital of USTC, Division of Life Sciences and Medicine, University of Science and Technology of China, Hefei, China; ^2^Neurodegenerative Disorder Research Center, Division of Life Sciences and Medicine, University of Science and Technology of China, Hefei, China; ^3^Department of Geriatric Medicine, The First Affiliated Hospital of USTC, Division of Life Sciences and Medicine, University of Science and Technology of China, Hefei, China

**Keywords:** biomarkers, serum complement, cognitive impairment, type-2 diabetes mellitus, metabolic disorders

## Abstract

**Background:**

The immune response and the complement system are associated with cognitive impairment and diabetes mellitus, respectively. Activation of the complement system in these diseases occurs mainly through either the classical pathway or the alternative pathway. However, the specific complement proteins involved in the development of the type 2 diabetes mellitus (T2DM) and cognitive impairment are still unclear. Here, we investigated complement proteins in serum from patients with T2DM, cognitive impairment, or both T2DM and cognitive impairment.

**Objective:**

To investigate the levels of serum immune complement proteins in patients with T2DM, cognitive impairment, or T2DM combined with cognitive impairment and the associations between these complement proteins and risk factors for T2DM or cognitive impairment.

**Methods:**

Clinical markers were collected from blood samples of 264 participants. Luminex multiplex assays were used to detect serum complement proteins. All statistical analyses were performed using Prism or R studio.

**Results:**

There was a difference in serum levels of the complement proteins C1q, C3, C3b, and FH between the three different groups. Hyperglycemia was significantly correlated with elevated C3b or reduced C3, C1q, and FH. In addition, hyperlipidemia was positively correlated with elevated levels of C3, C4, C1q, and FH proteins. There was an association between C1q, C3, C4, and FH and β-pancreas cell function, whereas only FH was associated with insulin resistance. Higher serum C1q was significantly associated with an increased risk of cognitive impairment.

**Conclusion:**

Serum levels of complement proteins were closely associated with hyperglycemia and hyperlipidemia. We found that classical complement pathway activation mainly occurred in the cognitive impairment only group, whereas the alternative pathway may reflect T2DM and T2DM with cognitive impairment.

## Introduction

Diabetes mellitus is ranked as one of the top 10 causes of death worldwide (Chatterjee et al., 2017). Type 2 diabetes mellitus (T2DM) is the most common type, accounting for 90% of all cases and is characterized by pancreatic β-cell dysfunction and insulin resistance (DeFronzo et al., 2015; Chatterjee et al., 2017). Individuals with T2DM are at a higher risk than non-diabetic individuals of developing dementia and cognitive impairment, such as Alzheimer’s disease (Biessels et al., 2006, 2014; Strachan et al., 2011; McCrimmon et al., 2012; Biessels and Reagan, 2015; Biessels and Despa, 2018), and between 10 and 15% of dementia cases worldwide may be attributed to T2DM (Biessels and Reagan, 2015). This poses a leading public threat to human health and to the worldwide economy, and as such, appropriate novel biomarkers for diagnosing or predicting Alzheimer’s disease are urgently needed.

A substantial body of evidence has documented that the complement system, an important arm of the innate immune system, is inextricably intertwined with the development of cognitive impairment and T2DM. A large volume of research has confirmed that the complement system is closely associated with insulin resistance (IR), β-cell function and diabetic vascular complications (Fujita et al., 2013; Ghosh et al., 2015; Flyvbjerg, 2017; Huang et al., 2018; Ajjan and Schroeder, 2019; Shim et al., 2020). A recent review reported that high complement protein C4A copy numbers and low C4B copy numbers are protective against residual β-cell function (Ajjan and Schroeder, 2019), and a clinical trial involving 95,202 participants has indicated that high baseline concentrations of complement C3 were associated with increased risk of diabetic neuropathy highlighted that complement component C3 has a role in the pathology of diabetic neuropathy (Rasmussen et al., 2018b). Complement also has a complex relationship with the CNS (Morgan and Harris, 2015; Hong et al., 2016; Coulthard et al., 2018; Morgan, 2018; Hammond et al., 2019; Lee et al., 2019; Reis et al., 2019; Dalakas et al., 2020); unbalanced or abnormal activation of complement can result in mental disorders, neurodevelopmental disorders or neurodegenerative diseases, including depression, schizophrenia, Alzheimer’s disease and Parkinson’s disease (Morgan and Harris, 2015; Hong et al., 2016; Sekar et al., 2016; Morgan, 2018; Hammond et al., 2019; Lee et al., 2019; Reis et al., 2019; Dalakas et al., 2020). For instance, a cohort study found that low baseline plasma levels of complement C3 were associated with a high risk of Alzheimer’s disease (Rasmussen et al., 2018a). Another clinical study showed that serum levels of C1q, another complement protein, were significantly higher in major depressive disorder patients than in controls (Yao and Li, 2020).

The complement system is an enzyme cascade involving multiple proteins and three different activating pathways: classical, lectin, and alternative. Activation of these different pathways may be associated with different diseases (Botto et al., 2009; Holers, 2014). The classical pathway is initiated by the early complement components C1 complex and C4. And the lectin pathway is activated when complement associated pattern recognition molecules, including MBL, ficolins and collectins bind to carbohydrate moieties on surfaces of pathogens.

One key regulator of the alternative pathway, a loop involving the assembly of the C3 convertase C3bBb, is the complement protein FH (Botto et al., 2009; Holers, 2014). However, the specific complement system activation pathway which is associated with T2DM, cognitive impairment and associated comorbidities is not well understood.

We conducted a cross-sectional study to prospectively determine the specific complement activating pathway, which includes different complement proteins (C1q, C3, C3b, C4, FH), in the pathology of T2DM only, cognitive impairment only, and T2DM combined with cognitive impairment. In addition, we also investigated the relationship between these complement proteins and several clinical risk factors and between cognitive or diabetic functional characteristics.

## Materials and Methods

### Study Participants

Our project included two separate cohorts. The first cohort was from the First Affiliated Hospital of The University of Science and Technology of China, and the second cohort was from the Provincial Sports Bureau Community, Hefei, Anhui, China. The demographics of the participants are described in Table 1. A total of 264 participants were enrolled in our study. We classified participants into four groups: normal (*n* = 70), T2DM only (*n* = 51), cognitive impairment only (*n* = 84) and T2DM combined with cognitive impairment (*n* = 59), predominantly according to fasting blood glucose levels (diabetes group if fasting blood glucose level ≥ 7 mmol/L) and scores on either Mini-Mental State Examination (MMSE) or Montreal Cognitive Assessment (MOCA) (cognitive impairment was defined as a score of ≤ 26 on either test). We did not consider other conditions that may fulfill the diagnostic criteria for T2DM or cognitive impairment.

**TABLE 1:** Demographic and clinical characteristics of participants.

	Total (*N* = 264)	Normal (*N* = 70)	T2DM (*N* = 51)	CI (*N* = 84)	T2DM&CI (*N* = 59)	*P*-value
**Sex**						
Female	129 (48.9%)	39 (55.7%)	15 (29.4%)	51 (60.7%)	24 (40.7%)	0.001
Male	135 (51.1%)	31 (44.3%)	36 (70.6%)	33 (39.3%)	35 (59.3%)	
Age						
Mean (SD)	63.0 (± 8.0)	64.0 (± 9.5)	61.1 (± 7.8)	63.2 (± 7.6)	63.2 (± 6.7)	0.26
**Complement proteins**						
C1q						
Mean (SD)	93.3 (± 21.3)	93.1 (± 20.4)	85.0 (± 18.1)	101.0 (± 21.2)	89.8 (± 21.6)	0.0001
C3						
Mean (SD)	60.9 (± 25.7)	62.6 (± 29.4)	55.9 (± 21.4)	66.4 (± 24.7)	55.5 (± 24.5)	0.033
C3b						
Mean (SD)	200.8 (± 159.3)	204.4 (± 169.3)	221.2 (± 177.9)	149.0 (± 122.5)	257.0 (± 158.5)	0.0007
C4						
Mean (SD)	214.2 (± 49.9)	210.2 (± 48.3)	210.4 (± 46.8)	224.1 (± 53.2)	208.2 (± 48.6)	0.18
FH						
Mean (SD)	247.0 (± 43.9)	251.8 (± 45.1)	238.6 (± 36.8)	255.6 (± 39.5)	236.2 (± 51.0)	0.022
**Clinical biochemical characteristics**						
BMI						
Mean (SD)	25.0 (± 11.7)	23.3 (± 2.9)	25.1 (± 3.1)	26.1 (± 19.5)	24.9 (± 3.4)	0.61
HDL						
Mean (SD)	1.2 (± 0.4)	1.3 (± 0.5)	1.0 (± 0.4)	1.3 (± 0.4)	1.0 (± 0.3)	<0.0001
LDL						
Mean (SD)	2.8 (± 0.9)	3.0 (± 0.9)	2.6 (± 0.9)	2.9 (± 0.8)	2.5 (± 0.9)	0.003
TC						
Mean (SD)	4.7 (± 1.3)	4.9 (± 1.0)	4.8 (± 2.1)	4.8 (± 0.9)	4.2 (± 1.2)	0.012
TG						
Mean (SD)	1.7 (± 1.1)	1.4 (± 0.6)	2.3 (± 1.8)	1.5 (± 0.6)	1.6 (± 1.0)	<0.0001
FBG						
Mean (SD)	6.3 (± 2.5)	4.9 (± 0.8)	7.9 (± 2.6)	5.0 (± 0.8)	7.9 (± 3.4)	<0.0001
FINS						
Mean (SD)	10.9 (± 10.3)	8.4 (± 4.3)	13.5 (± 17.9)	9.6 (± 4.9)	12.6 (± 9.2)	0.055
GHB						
Mean (SD)	8.1 (± 2.0)	6.6 (± 1.1)	9.9 (± 2.1)	6.8 (± 0.7)	9.3 (± 1.6)	<0.0001
HbA1C						
Mean (SD)	6.9 (± 1.9)	5.6 (± 0.4)	8.4 (± 2.0)	5.7 (± 0.6)	8.2 (± 1.7)	<0.0001
**Homeostasis model assessment characteristics**						
HOMO-IR						
Mean (SD)	3.1 (± 3.6)	1.9 (± 1.0)	4.8 (± 6.1)	2.2 (± 1.3)	4.0 (± 3.7)	<0.0001
HOMO-IS						
Mean (SD)	0.6 (± 0.4)	0.8 (± 0.6)	0.5 (± 0.4)	0.6 (± 0.4)	0.4 (± 0.2)	0.0002
HOMO-β						
Mean (SD)	97.1 (± 248.3)	135.9 (± 80.8)	90.0 (± 146.8)	142.3 (± 85.2)	0.7 (± 470.8)	0.017
IAI						
Mean (SD)	–3.9 (± 0.8)	–3.6 (± 0.6)	–4.2 (± 1.0)	–3.7 (± 0.6)	–4.3 (± 0.6)	<0.0001
**Cognitive functional characteristics**						
MMSE						
Mean (SD)	27.0 (± 3.2)	28.6 (± 1.1)	28.2 (± 1.7)	25.4 (± 4.0)	24.5 (± 3.6)	<0.0001
MOCA						
Mean (SD)	23.3 (± 4.4)	27.0 (± 1.1)	26.7 (± 1.1)	19.7 (± 3.4)	21.3 (± 4.4)	<0.0001

*FBG, fasting blood glucose; GHB, glycosylated hemoglobin; HbA1C, glycosylated hemoglobin A1c; HOMA-IR, homeostasis model assessment of insulin resistance; HOMA-IS, homeostasis model assessment of insulin sensitivity; HOMA-β, homeostasis model assessment of beta cell function index; IAI, insulin action index; HDL, high density lipoprotein; LDL, low density lipoprotein; TC, total cholesterol; TG, total triglyceride; HGB, hemoglobin; Ca, calcium; P, phosphate; MMSE, Mini-Mental State Examination; MOCA, Montreal Cognitive Assessment; FINS, fasting insulin; F, female; M, male. Data are presented as the median (standard deviation) where appropriate unless otherwise specified; p-values are derived from chi-square (sex) and Kruskal–Wallis tests (continuous variables).*

### Sample Preparation

Blood samples were collected from participants in the morning following an overnight fast. For each participant, blood was collected and centrifuged (2,000 g) for 10 min at room temperature (20–25°C) after allowing the blood to clot for 30 min–1 h. Following centrifugation, serum from all tubes were transferred into 200 μL aliquots, put into 1.5-mL polypropylene protein low-binding tubes which were placed immediately on dry ice and stored at -80°C. Before assays were performed, samples were thawed on ice, and aliquots of 20 μL were transferred into 1.5-mL polypropylene protein low-binding tubes and stored at -80°C. When using these samples, we placed them on ice and thawed again, such that the samples used in our assays had only two freeze cycles to avoid complement activation.

### Detection of Serum Complements

Luminex multiplex assays were used to detect all five complement proteins, C1q, C3, C3b, C4, and FH. Serum samples were thawed on ice until assay application. Serum standards and backgrounds were run in duplicate. Following the manufacturer instructions for the human complement magnetic bead panel-2 kit (cat. #HCMP2MAG-19K, Merck, Germany), we diluted serum at 1:8,000 with assay buffer and incubated overnight on a plate shaker with agitation (16–18 h) at 4°C after adding magnetic beads. Detection antibodies were incubated with agitation on a plate shaker for 1 h at room temperature (20–25°C), and streptavidin-phycoerythrin was then incubated for 30 min. After incubation, beads were resuspended using sheath fluid (Luminex Corporation, TX, United States), and median fluorescent intensity (MFI) values were read and analyzed using a Luminex^®^ 200™ system (Luminex Corporation, TX, United States).

### Statistical Analysis

**FIGURE 1:**
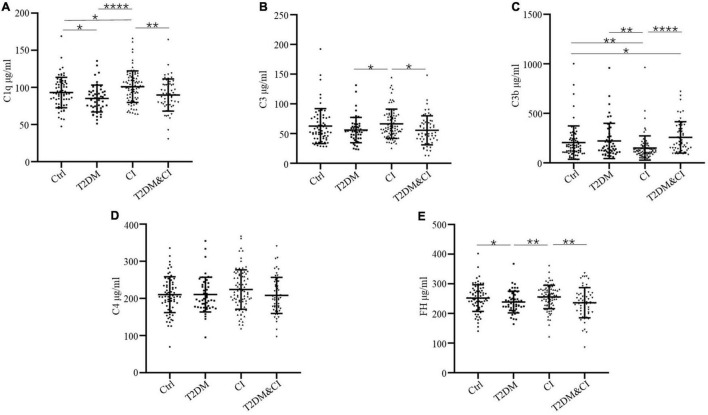
Serum levels of complement proteins in the cross-sectional control (Ctrl), diabetes mellitus only (T2DM), cognitive impairment only (CI) and diabetes combined with cognitive impairment groups (T2DM&CI). Each point represents the value for an individual control or participant, and the horizontal line in point clusters represents the mean level and standard deviation for that group. Bar graphs indicate statistical significance of differences in values for each two-group comparison, calculated by Mann–Whitney *U*-test pairwise comparisons (*p < 0.05; **p < 0.01; ***p < 0.005; ****p < 0.0001). See Table 1 for statistical details. **(A)** C1q, **(B)** C3, **(C)** C3b, **(D)** C4, **(E)** FH.

Data analyses and visualization were performed using Prism 8 (GraphPad Software Inc., La Jolla, CA) and R studio. Medians and standard deviations (SD) were calculated for demographic characteristics as described in Table 1 and Figure 1. Normal data distributions were assessed using the Shapiro–Wilk test and Box-Cox transformation (log-transformed) was applied to non-normal data following the removal of any outliers. Non-parametric comparisons of complement protein concentrations between groups were performed using Mann–Whitney *U*-tests. Age was not adjusted for because there was no significant age difference between groups.

For other clinical characteristics, Kruskal–Wallis tests were used to compare continuous variables between groups, and chi-square tests (χ^2^-test) were used to compare dichotomous variables. Specifically, for serum levels of complement proteins: C3 data were log-transformed; C3b data were log-transformed and five outliers were removed; six outliers were removed from C1q data, and four outliers were removed from C4 data. For HOMA index calculations, HOMA-IR values were log-transformed, and 7 outliers were then removed. Eight outliers were removed from the HOMA-β values, and then HOMA-β values were transformed to [HOMA-β]^–0^.^5^. And seven outliers were removed from the insulin action index (IAI) values.

Associations between serum complement proteins and other clinical characteristics and fasting blood sugar levels were examined using linear regression models either adjusted for age and sex or not adjusted, as described in Tables 2, 3. In the linear regression models, the β coefficient for the risk factors represents the cross-sectional association with the serum complement levels. In addition to examining the associations with clinical characteristics and serum complement proteins, we sought to investigate the association between complement levels and cognitive impairment using generalized linear models, either adjusted for age and sex or not adjusted (see Table 3). A positive odds ratio (OR) indicated that an improvement in the serum levels of complement proteins was associated with an increased risk of cognitive impairment.

**TABLE 2:** Associations of complement protein with clinical parameters.

	C3b		C3		C1q		C4		FH	
	Unstandardized estimate (SE)	*P*-value	Unstandardized estimate (SE)	*P*-value	Unstandardized estimate (SE)	*P*-value	Unstandardized estimate (SE)	*P*-value	Unstandardized estimate (SE)	*P*-value
**Adjusted for sex and age**										
BMI	0.0012 (0.0033)	0.71	0.001 (0.0022)	0.66	0.0689 (0.1173)	0.56	0.2207 (0.2752)	0.42	0.2144 (0.2324)	0.36
FBG	0.0402 (0.016)	0.013^a^	-0.0212 (0.01)	0.034^a^	-1.4775 (0.5453)	0.0072^b^	-1.5446 (1.2994)	0.24	-2.3995 (1.0747)	0.026^a^
GHB	0.0793 (0.0195)	<0.0001^c^	-0.0062 (0.0136)	0.65	-2.6053 (0.6883)	0.0002^c^	-0.7675 (1.6239)	0.64	-2.4829 (1.4209)	0.082
HbA1C	0.0734 (0.0216)	<0.0001^c^	-0.0137 (0.0147)	0.35	-2.7452 (0.7483)	0.0003^c^	-1.6784 (1.7594)	0.34	-3.0892 (1.5385)	0.046^a^
HDL	-0.2623 (0.0983)	0.0082^b^	0.002 (0.0672)	0.98	2.6561 (3.6209)	0.46	-2.4891 (8.3662)	0.77	-6.7959 (7.2062)	0.35
LDL	-0.057 (0.0443)	0.20	0.0968 (0.0288)	0.0009^c^	3.7406 (1.5742)	0.018^a^	8.6559 (3.6348)	0.018^a^	11.1083 (3.1002)	0.0004^c^
TC	-0.0133 (0.0295)	0.65	0.0465 (0.0195)	0.018^a^	1.9655 (1.057)	0.064	3.4541 (2.4484)	0.16	3.0574 (2.1191)	0.15
TG	0.0522 (0.034)	0.13	0.0362 (0.0232)	0.12	2.9051 (1.2432)	0.02^a^	1.0342 (2.9034)	0.72	4.8482 (2.4938)	0.053
**Not adjusted for sex and age**										
BMI	0.0006 (0.0033)	0.87	0.0018 (0.0023)	0.44	0.0875 (0.1197)	0.47	0.2636 (0.2761)	0.34	0.243 (0.2354)	0.30
FBG	0.046 (0.0154)	0.0031^b^	-0.0338 (0.01)	0.0008^c^	-1.8695 (0.5304)	0.0005^c^	-2.249 (1.252)	0.074	-3.123 (1.044)	0.0031^b^
GHB	0.0846 (0.019)	<0.0001^c^	-0.0186 (0.0137)	0.18	-3.0719 (0.6801)	<0.0001^c^	-1.599 (1.596)	0.32	-3.341 (1.398)	0.018^a^
HbA1C	0.0795 (0.021)	0.0002^c^	-0.0273 (0.0148)	0.067	-3.2713 (0.7375)	<0.0001^c^	-2.556 (1.724)	0.14	-4.029 (1.509)	0.0082^b^
HDL	-0.2856 (0.094)	0.0027^b^	0.0783 (0.0664)	0.24	6.223 (3.504)	0.077	2.663 (8.008)	0.74	0.5837 (6.9859)	0.93
LDL	-0.0581 (0.0439)	0.19	0.1043 (0.0295)	0.0005^c^	4.492 (1.576)	0.0048^b^	9.211 (3.593)	0.011^a^	12.275 (3.081)	<0.0001^c^
TC	-0.0193 (0.029)	0.51	0.0573 (0.0197)	0.004^b^	2.66 (1.049)	0.012^a^	4.193 (2.399)	0.082	4.278 (2.092)	0.042^a^
TG	0.0587 (0.0342)	0.087	0.0277 (0.0241)	0.25	2.709 (1.27)	0.034^a^	0.5836 (2.9122)	0.84	4.527 (2.528)	0.075

*BMI, body mass index; FBG, fasting blood glucose; FINS, fasting serum insulin; GHB, glycosylated hemoglobin; HbA1C, glycosylated hemoglobin A1c; HDL, high-density lipoprotein; LDL, low-density lipoprotein; TC, total cholesterol; TG, total triglycerides; HGB, hemoglobin.*

*The results were obtained using linear regression models with or without adjustment for age and sex. Numbers presented are unstandardized estimates (SEs) and P-values for the association of each independent variable with complement proteins. Some outliers were removed from dependent variable data or Box–Cox transformed for normalization.*

***^a–c^**Indicates a statistically significant difference: p < 0.05, p < 0.01, p < 0.001.*

**TABLE 3:** Associations of insulin resistance or cognitive function with complement proteins. **(A)** Linear regression analysis assessing complement proteins associated with insulin resistance and β-cell function. **(B)** Logistic regression analysis assessing complement proteins associated with cognitive impairment.

	HOMA-IR		HOMA-β		IAI	
	Unstandardized estimate (SE)	*P*-value	Unstandardized estimate (SE)	*P*-value	Unstandardized estimate (SE)	*P*-value
**A**						
**Adjusted for sex and age**						
C1q	0.0024 (0.0023)	0.29	0.0377 (0.0124)	0.0026^b^	-0.0019 (0.0026)	0.46
C3	0.0024 (0.0019)	0.19	0.0235 (0.0103)	0.023^a^	-0.0026 (0.0021)	0.22
C3b	0.0004 (0.0003)	0.22	-0.001 (0.0017)	0.57	-0.0005 (0.0004)	0.20
C4	0.0017 (0.001)	0.088	0.0134 (0.0054)	0.013^a^	-0.0017 (0.0011)	0.12
FH	0.0036 (0.0011)	0.0018^b^	0.0195 (0.0064)	0.0028^b^	-0.0035 (0.0013)	0.0071^b^
**Not adjusted for sex and age**						
C1q	0.0013 (0.0022)	0.57	0.0443 (0.0119)	0.0003^c^	-0.0002 (0.0025)	0.95
C3	0.0013 (0.0018)	0.46	0.03 (0.0099)	0.0027^b^	-0.0009 (0.002)	0.65
C3b	0.0005 (0.0003)	0.13	-0.0018 (0.0017)	0.30	-0.0006 (0.0004)	0.083
C4	0.0014 (0.001)	0.17	0.016 (0.0053)	0.0029^b^	-0.0013 (0.0011)	0.24
FH	0.0029 (0.0011)	0.0096^b^	0.0229 (0.0062)	0.0003^c^	-0.0025 (0.0013)	0.053

**B**						
	**Model1^d^**			**Model2**		
	**OR**	**95%CI**	***P*-value**	**OR**	**95%CI**	***P*-value**

C1q	1.0146	(1.0024, 1.0275)	0.021^e^	1.0155	(1.0036, 1.0282)	0.012^e^
C3	1.0017	(0.9919, 1.0118)	0.74	1.0033	(0.9938, 1.0131)	0.50
C3b	0.9994	(0.9978, 1.0010)	0.45	0.9992	(0.9976, 1.0008)	0.32
C4	1.0027	(0.9978, 1.0077)	0.29	1.0030	(0.9981, 1.0080)	0.24
FH	1.0002	(0.9946, 1.0059)	0.94	1.0007	(0.9952, 1.0063)	0.80

*HOMA-IR, homeostasis model assessment of insulin resistance; HOMA-β, homeostasis model assessment of beta cell function index; IAI, insulin action index.*

*The results were obtained using linear models with or without adjustment for age and sex. Numbers presented are estimates (SEs) and P-values for the association of each complement protein with homeostasis model assessment. Some dependent variables were Box–Cox transformed, and/or some observation points were removed for normalization.*

***^a–c^**Indicates a statistically significant difference: p < 0.05, p < 0.01, p < 0.001.*

*OR, odds ratio. The results were obtained using logistic regression analysis with or without adjustment for age and sex. Numbers presented are ORs and P-values for the association of each complement protein with cognitive status.*

***^d^**Adjusted for age and sex.*

***^e^**Indicates a statistically significant difference: p < 0.05.*

Statistical significance was indicated by *p* < 0.05.

## Results

### Comparison of Serum Complement Protein Levels Across All Groups

264 participants were enrolled in our study. Serum levels of C1q, C3, C3b, C4, and FH across each group are shown in Table 1 and Figure 1. Other demographic and clinical characteristics are summarized in Table 1. Classification criteria for group assignment are described in the methods.

Levels of C3b in both the T2DM and CI groups were significantly different to healthy controls (mean ± SD, 257.0 ± 158.5 μg/mL for the T2DM&CI group and 204.4 ± 169.3 μg/mL for control group, *p* < 0.05). There were no other differences in protein levels between these groups.

With the exception of C4, there were significant differences in all complement protein levels measured between the T2DM and CI groups. There were significantly higher levels of C1q, C3, and FH in the CI group than in the T2DM group (*p* < 0.0001 for C1q, *p* < 0.05 for C3 and *p* < 0.01 for FH). Conversely, mean serum levels of C3b were higher in the T2DM group than in the CI group (*p* < 0.01). In the T2DM&CI group, C3b levels were significantly higher than in the CI group (*p* < 0.0001).

However, there were no differences in mean serum level of any complement proteins between the T2DM and T2DM&CI groups. In contrast to the above results, mean serum levels of C4 across all patients were indistinguishable from controls (Figure 1).

### Association of Levels of Complement Proteins With Clinical Parameters

Consistent with the above findings, we found that clinical characteristics were also different between all groups (Table 1). We hypothesized that levels of complement proteins would be associated with the hyperglycemia and hyperlipemia indicators. This hypothesis was tested using single factor linear regression models. Full model statistics for all analyses are reported in Table 2, which shows that FBG (Fasting blood sugar), GHB (glycated hemoglobin), HbA1C (glycosylated hemoglobin A1c) were significantly associated with higher levels of C3b [β(SE), 0.0402(0.016), *p* = 0.013 for FBG; β(SE), 0.0793(0.0195), *p* < 0.0001 for GHB; β(SE), 0.0734(0.0216),*p* < 0.0001 for HbA1C], and HDL (High density lipoprotein) was significantly associated with lower levels of C3b [β(SE), ^®^ 0.2623(0.0983), *p* = 0.0082]. Similar results were found when the data were adjusted for sex and age [β(SE), 0.046(0.0154), *p* = 0.0031 for FBG; β(SE), 0.0846(0.019), *p* < 0.0001 for GHB; β(SE), 0.0795(0.021), *p* < 0.0002 for HbA1C; β(SE), -0.2856(0.094), *p* = 0.0027 for HDL]. We observed a significant negative relationship between C3 and FBG [β(SE), -0.0212(0.01), *p* = 0.034] and a significant positive relationship between C3 and both LDL (Low density lipoprotein) and TC (Total cholesterol) [β(SE), 0.0968(0.0288), *p* < 0.0009 for LDL; β(SE), 0.0465(0.0195), *p* = 0.018 for TC] (Table 2).

We also found that higher levels of C1q were significantly associated with both lower levels of FBG, GHB or HbA1C and greater LDL, TC or TG [β(SE), -1.4775(0.5453), *p* < 0.0072, for FBG; β(SE), -2.6053(0.6883), *p* < 0.0002, for GHB; β(SE), -2.7452(0.7483), *p* < 0.0003, for HbA1C, β(SE), 3.7406(1.5742), *p* = 0.018, for LDL; β(SE), 1.9655(1.057), *p* = 0.064, for TC; β(SE), 2.9051(1.2432), *p* = 0.02, for TG, respectively]. These associations were also statistically significant after adjusting for age and sex, with the exception of the association between C1q and TC. Similarly, FBG, GHB and HbA1c were significantly associated with lower levels of the complement protein FH [β(SE), -2.3995 (1.0747), *p* = 0.026, for FBG; β(SE), -2.4829(1.4209), *p* = 0.082, for GHB; β(SE), -3.0892(1.5385), *p* = 0.046, for HbA1c]. Conversely, there was a significant positive association between FH with both LDL and TC and [β(SE), 11.1083(3.1002), *p* < 0.0004, for LDL; β(SE), 3.0574(2.1191), *p* = 0.15, for TC]. We found that β estimates for C4-LDL positive associations were significant whether or not the data was adjusted for age and sex (*p* = 0.018, adjusted for age and sex; *p* = 1.013, not adjusted for age and sex) (Table 2).

### Association of Serum Complement Protein Levels With Cognitive Status and Homeostasis Model Assessment

Next, to investigate the functions of complement proteins in pathology, including cognitive impairment and insulin resistance, we derived an estimate of the association between complement proteins and insulin resistance or cognitive function.

First, to better understand the associations between complement proteins and homeostatic model assessment, including HOMA-IR, HOMA-β and IAI, we conducted linear regression analyses (full model statistics are presented in Table 3). We found that the complement regulator FH was significantly associated with HOMA-IR and HOMA-β. However, there was a significant negative relationship between FH and IAI when adjustments for age and sex were made [β(SE), -0.0035 (0.0013), *p* < 0.0071]. Complement proteins C1q, C3 and C4 were only significantly associated with HOMA-β, an index of islet beta cell function [β(SE), 0.0377 (0.0124), *p* = 0.0026, for C1q; β(SE), 0.0235 (0.0103), *p* = 0.023, for C3; β(SE), 0.0134 (0.0054), *p* = 0.013, for C4]. Without adjusting for age and sex, there was no association between FH and IAI [β(SE), -0.0025 (0.0013); *p* = 0.053]. In summary, we found associations between increased levels of complement proteins, particularly FH, and insulin resistance.

We further examined the relationship between the levels of complement proteins and cognitive function using general linear models. To obtain a better understanding of the relationships between complement proteins and the cognition, we stratified individuals according to the presence of cognitive impairment (i.e., impaired cognition vs. normal cognition). We found a statistically significant relationship between cognitive group and C1q. The estimated odds ratio (OR) for dementia was significantly higher in participants with elevated C1q (odds ratio (OR), 1.0155; (95% CI, 1.0036–1.0282); *P* = 0.012), even after controlling for age and sex (odds ratio (OR), 1.0146 (95% CI, 1.0024–1.0275); P = 0.021). This indicates that individuals with higher serum levels of C1q tended to have a greater likelihood of cognitive impairment (Table 3). No other complement protein was associated with cognitive group.

## Discussion

In this study, we found that there were significant differences between patients with cognitive impairment only, T2DM, or T2DM with cognitive impairment in the levels of the complement proteins C1q, C3, C3b and the complement regulatory protein FH (Figure 1). These results suggest that there are different complement activation pathways underlying cognitive impairment without T2DM and cognitive impairment with T2DM.

First, serum C1q was significantly higher in individuals with cognitive impairment only than in individuals with cognitive impairment and T2DM. Previous work has revealed detrimental effects of C1q in neuronal injury and nervous system disease through the promotion of amyloid plaque accumulation, phosphorylated neurofibrillary tangles and the exacerbation of neuroinflammation (Hong et al., 2016; Morgan, 2018; Lee et al., 2019). In addition, studies have demonstrated that the classical pathway complement proteins, C1q, C3, C4 in particular, are involved in the pathology of neurological diseases, including Alzheimer’s disease, through the facilitation Aβ clearance or synapse engulfment by reactive microglia (Loeffler, 2004; Propson et al., 2021). Moreover, C1q activates the complement proteins C1r and C1s by binding to Ig-G and Ig-M, resulting in the cleavage of downstream C4 and activation of the classical pathway (Sarma and Ward, 2011). However, there was no difference in serum C4 between the groups in the present study. Many recent studies have found that C4 is related to central nervous system disorders, particularly schizophrenia (Sarma and Ward, 2011). However, no study has found a link between C4 and cognitive impairment. Thus, it is likely that C4 is not involved in the pathogenesis of cognitive impairment (without T2DM).

The activation of the classical complement pathway may come from the actived astrocytes and microglia. Previous research has also shown that neuroinflammation, activation of astrocytes and microglia are hallmarks of the neurological diseases and cognitive impairment. And the reactived astrocytes and microglia secrete C3 and C1q (Propson et al., 2021). Meanwhile other pathological characteristics of the neurodegeneration, such as Aβ and hyperphosphorylated tau could also increase the levels and depositions of C1q as well (Hong et al., 2016; Schartz and Tenner, 2020). These compelling studies may help inspire thinking for the activation mechanism of the classical complement pathway.

Interestingly, serum levels of C3b, a byproduct of the alternative pathway, were lower in the cognitive impairment only group. C3b is an integral part of the C3 transformation enzyme C3Bb, which enhances the activation stage of the alternative pathway (Ghosh et al., 2015). This indicates that the alternative pathway was inhibited in the cognitive impairment only group. Studies have also shown that C3b plays an important role in the nervous system. On the one hand, it promotes brain maturation through synaptic conditioning, neuron migration and synaptic trimming to promote complete functional brain maturity (Coulthard et al., 2018; Lee et al., 2019; Reis et al., 2019) and on the other hand, C3b/iC3Bb is also deposited around the damaged brain (Lee et al., 2019; Reis et al., 2019). However, the C3 protein and other byproducts, such as C3d, are considered to be closely related to diabetes and its complications, although there is limited research on C3b and diabetes (Ajjan and Schroeder, 2019). The present study found that the serum levels of C3b were highest in the comorbid T2DM group, indicating that the activation of the alternative pathway may play a key role in T2DM-related cognitive disorder.

The main function of the component protein FH is to inhibit the formation of C3 transformase C3bBb, thereby avoiding excessive activation of the complement system (Botto et al., 2009). Clinical studies have demonstrated that FH is higher in individuals with insulin resistance, metabolic dysfunction, and obesity (Moreno-Navarrete and Fernandez-Real, 2019; Shim et al., 2020). Our current study found that serum FH was lower in the T2DM group than in the control group, which may contribute to the activation of the alternative pathway in T2DM.

Meanwhile, the alternative complement pathway is activated in obese T2DM individuals. Many studies have also demonstrated that adipose tissue is a major site of synthesis of the necessary components for alternative complement pathway (Vlaicu et al., 2016; King and Blom, 2021). Moreover, components of the alternative pathway are secreted from the activated adipocyte as well as the hepatocyte under obesogenic and hyperlipidaemic conditions in obese T2DM patients, and are especially induced by post-prandial hyperchylomicronaemia (Fujita et al., 2013). The overexpression of the complement proteins also leads to positive feedback of complement activation, expression and related pathology (King and Blom, 2021).

In our study, we found that the classical complement pathway underlies cognitive impairment and the alternative complement pathway underlies T2DM pathology when combined with cognitive impairment. The mechanisms of the different complement activation pathways stem from different pathologic features of different types of cognitive impairment. In detail, the chronic metabolic disease T2DM and the activation of the alternative complement pathway in cognitive impaired individuals with T2DM is likely due to T2DM pathology, especially as it is closely related to the obese and activated fat cells.

In this study, we also observed that blood glucose indicators, including FBG, GHB, and HbA1c, were associated with serum levels of complement proteins. Hyperglycemia was associated with lower levels of C1q, C3, and FH but higher serum levels of C3b. This suggests that hyperglycemia may cause activation of the classical pathway and inhibit the alternative pathway. These results are consistent with some cross-sectional and longitudinal observations between plasma levels of complement protein and high blood sugar (Wlazlo et al., 2014; Borne et al., 2017). Moreover, we observed that higher LDL was correlated with higher serum levels of C1q, C3, C4 and FH. This thus suggests that hyperlipidemia may also result in activation of the classical pathway and inhibition of the alternative pathway. Contrary results suggest that hyperlipidemia and hyperglycemia may affect complement activation through different pathways (Jones, 2016), although there is a close relationship between glucose metabolism and lipid metabolism (Jones, 2016).

T2DM is a metabolic disorder characterized by chronic hyperglycemia and hyperlipidemia (DeFronzo et al., 2015; Chatterjee et al., 2017). Hyperglycemia may induce tissue damage typical of diabetic complications (Ghosh et al., 2015) and increase the hazard ratio for dementia (Crane et al., 2013). In addition, neurodegenerative diseases, especially AD, are often accompanied by symptoms of energy metabolism imbalance (Kapogiannis and Mattson, 2011). In the present study, we reveal a significant relationship between the elevated complement proteins C1q, C3, C4, and FH and enhanced β-cell function (HOMA-β) and insulin resistance (HOMA-IR). In particular, FH, which is involved in the alternative pathway, may be a vital independent risk factor for insulin resistance [β(SE), 0.0195 (0.0064), *P* = 0.0028]. Additionally, we found that patients with higher C1q had a higher level of cognitive dysfunction (when adjusted for sex and age; odds ratio, 1.0146 (95% CI, 1.0024–1.0275), *P* = 0.021), which indicates that serum C1q could be a useful novel early warning sign for cognitive impairment; other work has confirmed that C1q is associated with cognitive impairment (Stevens et al., 2007; Hong et al., 2016; Morgan, 2018; Cho, 2019; Hammond et al., 2019). These results indicate that abnormal blood glucose and lipids may be a risk factor affecting serum complement protein levels and activation of the complement pathway.

Moreover, there is a strong link between diabetes and cognitive impairment; the risk for dementia is higher in T2DM patients than in those without T2DM (Biessels et al., 2006; Biessels and Despa, 2018). The ability of some drugs, such as metformin, which is used to in the treatment of T2DM to lower blood glucose and also improve cognitive decline, also confirm the relationship between neuro-psychiatric disorders and metabolic disorders (Markowicz-Piasecka et al., 2017). However, the specific mechanism between T2DM and cognitive impairment is still not clear. Some studies have demonstrated that insulin resistance in the CNS is a mechanistic mediator of structural brain and cognitive deficits *via* inflammation, oxidative stress and direct cellular effects (Biessels and Reagan, 2015; Arnold et al., 2018). In our present study, we verified that complement activation may underlie the pathology of T2DM and cognitive impairment. This is consistent with previous evidence showing that complement proteins accumulate in the focal areas associated with T2DM or neurodegenerative disorders and consistent with several clinical experiments showing the involvement of serum complement proteins (Fujita et al., 2013; Ghosh et al., 2015; Morgan and Harris, 2015; Hong et al., 2016; Ajjan and Schroeder, 2019; Hammond et al., 2019; Lee et al., 2019; Shim et al., 2020). In addition, there is evidence that complement enrichment may cause blood–brain barrier damage and cognitive impairment *via* apoptosis of brain endothelial cells, causing infiltration of inflammatory cells and consequent opening of tight junction constructs, which modulate the generation of cytokines and chemokines (Veerhuis et al., 2011; Jacob and Alexander, 2014; Alexander, 2018; Morgan, 2018; Dalakas et al., 2020; Propson et al., 2021). We found a difference in serum levels of complement proteins between the comorbidity group and cognitive impairment group, which indicates that complement proteins cause different types of nerve injury through different activation pathways. Higher serum levels of C1q, C3 and FH in the cognitive impairment only group indicate that activation of the classical pathway and inhibition of the alternative pathway underlie the neuronal damage within this group. Conversely, activation of the alternative pathway results in T2DM-related cognitive damage.

In summary, we found different serum levels of complement proteins between patient groups and found that were risk factors for these changes. We further determined the specific complement activating pathways underlying different types of neurotoxicity in patients with cognitive impairment only and T2DM-related cognitive damage. Our findings are limited since they are based solely on mathematical models; however, they use data from a relatively large pool of individuals. Future longitudinal investigations using larger participant populations are required to validate our original findings.

## Limitations

Given the nature of our study design, care needs to be taken with interpretation. First, there were no other physical diagnostic tools used as evidence of cognitive function. Neuroimaging or CSF biomarkers would permit more definitive interpretation. Second, we did not determine whether the type of cognitive impairment we measured was a diabetic complication. Long-term follow-up cohorts may verify this in the future. Third, participants in the study were primarily Anhui people and cannot adequately reflect the racial and ethnic diversity of China or the Chinese population, even the larger global population. Participants from other regions and global multi countries will need to be included in the future. Moreover, potential confounding variables that may affect cognitive function or serum complement levels, such as unhealthy lifestyle or past disease history, were not accounted for. As such, there is much groundwork to be done before we can exploit the potential role of complement proteins as prognostic markers.

## Conclusion

As noted above, we present new evidence that pathology relating to insulin resistance and cognitive dysfunction may be induced by different complement activating pathways in a manner dependent on abnormal blood glucose and lipid fluctuations. Given the growing morbidity of individuals with T2DM and dementia, our complement protein findings may help diagnose, predict or stratify T2DM and cognitive impairment early and accurately.

## Data Availability Statement

The original contributions presented in the study are included in the article/supplementary material, further inquiries can be directed to the corresponding author/s.

## Ethics Statement

The studies involving human participants were reviewed and approved by the Ethics Committee of Anhui Provincial Hospital Medical Research (approval #89). Written informed consent for participation was not required for this study in accordance with the national legislation and the institutional requirements.

## Author Contributions

ZL: acquisition, analysis, interpretation of data, drafting of the manuscript, and statistical analysis. ZL and WZ: collection of samples. FG and YS: critical revision of the manuscript for important intellectual content. YS, DK, and QT: funding acquisition and supervision. All authors have contributed to the work and agree with the presented results.

## Conflict of Interest

The authors declare that the research was conducted in the absence of any commercial or financial relationships that could be construed as a potential conflict of interest.

## Publisher’s Note

All claims expressed in this article are solely those of the authors and do not necessarily represent those of their affiliated organizations, or those of the publisher, the editors and the reviewers. Any product that may be evaluated in this article, or claim that may be made by its manufacturer, is not guaranteed or endorsed by the publisher.
